# A predictive model for preterm infants with bronchopulmonary dysplasia based on ferroptosis-related lncRNAs

**DOI:** 10.1186/s12890-023-02670-7

**Published:** 2023-10-02

**Authors:** Ziming Zhang, Kewei Chen, Dandan Pan, Tieshuai Liu, Chengcheng Hang, Yuhan Ying, Jia He, Ying Lv, Xiaolu Ma, Zheng Chen, Ling Liu, Jiajun Zhu, Lizhong Du

**Affiliations:** 1 Neonatal Intensive Care Unit, Children’s Hospital, Zhejiang University School of Medicine, National Clinical Research Center for Child Health, Hangzhou, China; 2 Department of Neonatology, Children’s Hospital, Zhejiang University School of Medicine, National Clinical Research Center for Child Health, Hangzhou, China; 3 Department of Neonatology, Guiyang Maternal and Child Health Care Hospital, Guiyang, China; 4 Department of Anesthesiology, Sir Run Run Shaw Hospital School of Medicine, Zhejiang University, Hangzhou, China; 5 Teaching Experimental Center of Public Health, Zhejiang University, Hangzhou, China; 6 Department and Child Health Care, Children’s Hospital, Zhejiang University School of Medicine, National Clinical Research Center for Child Health, Hangzhou, China; 7 Department of Neonatology, Women’s Hospital School of Medicine, Zhejiang University, Key Laboratory& Women’s Hospital, Hangzhou, China

**Keywords:** Bronchopulmonary dysplasia, LncRNA, Ferroptosis, Preterm infant, Diagnosis

## Abstract

**Background:**

Bronchopulmonary dysplasia (BPD) is the most challenging chronic lung disease for prematurity, with difficulties in early identification. Given lncRNA emerging as a novel biomarker and the regulator of ferroptosis, this study aims to develop a BPD predictive model based on ferroptosis-related lncRNAs (FRLs).

**Methods:**

Using a rat model, we firstly explored mRNA levels of ferroptosis-related genes and ferrous iron accumulation in BPD rat lungs. Subsequently, a microarray dataset of umbilical cord tissue from 20 preterm infants with BPD and 34 preterm infants without BPD were downloaded from the Gene Expression Omnibus databases. Random forest and LASSO regression were conducted to identify diagnostic FRLs. Nomogram was used to construct a predictive BPD model based on the FRLs. Finally, umbilical cord blood lymphocytes of preterm infants born before 32 weeks gestational age and term infants were collected and determined the expression level of diagnostic FRLs by RT-qPCR.

**Results:**

Increased iron accumulation and several dysregulated ferroptosis-associated genes were found in BPD rat lung tissues, indicating that ferroptosis was participating in the development of BPD. By exploring the microarray dataset of preterm infants with BPD, 6 FRLs, namely LINC00348, POT1-AS1, LINC01103, TTTY8, PACRG-AS1, LINC00691, were determined as diagnostic FRLs for modeling. The area under the receiver operator characteristic curve of the model was 0.932, showing good discrimination of BPD. In accordance with our analysis of microarray dataset, the mRNA levels of FRLs were significantly upregulated in umbilical cord blood lymphocytes from preterm infants who had high risk of BPD.

**Conclusion:**

The incorporation of FRLs into a predictive model offers a non-invasive approach to show promise in improving early detection and management of this challenging chronic lung disease in premature infant, enabling timely intervention and personalized treatment strategies.

## Background

Bronchopulmonary dysplasia (BPD) is a chronic respiratory complication most commonly occurring in premature infants [1]. With an incidence up to 45% in infants born before 29 weeks of gestational age, it stands as a major contributor to preterm morbidity and mortality [2]. Despite improvement in neonatal medicine, the incidence of BPD has continued to rise in recent decades [3]. Moreover, it is not merely a lung disease, but also a systemic condition with through-life impact. Infants with BPD are at high risk of lifelong pulmonary vascular and airway disease, as well as poor cognitive development [4–6]. Early identification of BPD in preterm infants enables timely treatment to reduce adverse effects and improve clinical outcomes. However, the early recognition poses challenges due to its substantial heterogeneity in clinical presentation, which could be influenced by positive pressure, medications and supplemental oxygen [7]. According to the National Institute of Child Health and Human Development (NICHD) diagnosis criteria, the diagnosis could not be finished until postmenstrual age of 36 weeks [1]. Therefore, there is a pressing need to explore biomarkers of BPD.

Ferroptosis, a newly defined form of cell death characterized by iron-dependent lipid peroxidation, is closely associated with the occurrence and progression of various respiratory disease, including acute respiratory distress syndrome (ARDS), asthma and acute lung injury [8–10]. Intriguingly, an increasing amount of evidence suggests that it is implicated in pathological development of BPD in recent years [11, 12]. A clinical study has revealed elevated free iron concentration in umbilical cord blood of premature infants compared to full-term infants, with cumulative enteral iron increasing the risk of BPD [13]. Abnormal iron accumulation has also been observed in lungs from BPD mice, and inhibiting ferroptosis has shown promise in attenuating hyperoxia-induced lung injury [14].

Long noncoding RNAs (lncRNAs) are functional RNA transcripts with a length of more than 200 nucleotides. They affect expression of target genes at multiple levels, including transcription, translation, and post-translation, by binding to DNA, RNA, and proteins [15]. Moreover, lncRNAs have been identified as key regulators in the ferroptosis pathway [16]. For instance, lncRNA P53RRA induced ferroptosis via nuclear sequestration of p53 [17]. LINC00578 suppressed ferroptosis by decreasing solute carrier family 7 member 11 (SCL7A11) ubiquitination [18]. Ferroptosis-related lncRNAs become valuable additions to the repertoire of prognostic tools in respiratory diseases, such as lung carcinomas and asthma [19, 20]. A total of 10 FRLs, namely RP11-386M24.3, LINC00592, FENDRR, AC104699.1, AC091132.1, LANCL1-AS1, LINC-PINT, IFNG-AS1, LINC00968 and AC006129.2, were identified as independent predictors of lung adenocarcinoma outcome [21]. However, FRLs for BPD prediction remained largely unknown. This study aims to identify diagnostic FRLs and construct a predictive model for early recognition of BPD.

## Materials and methods

### Establishment of BPD rat model

The lungs of term newborn rats were structurally similar to the those of extremely preterm infants [22], so that neonatal rats expose to hyperoxia were widely used to establish the BPD animal model. All methods were carried out in accordance with guidelines and regulations of the Institutional Animal Care and Use Committee of Zhejiang University. All methods were in accordance with ARRIVE guidelines (https://arriveguidelines.org) for the animal experiments. Sprague–Dawley rats were purchased and raised with a 12-h light–dark cycle. Pregnant Sprague–Dawley rat were raised in individual cages with free access to food and water. Within 8 h of birth, the pups were randomly assigned to two parts. The pups were reared in room air for 21 days named control group. The others reared in a plexiglass chamber with the fraction of oxygen (85%) for 21 days named BPD group. To prevent oxygen toxicity of the mother rats, the mother rats were rotated every 24-h between the oxygen treatment and the control litters. On postnatal day 21, all the pups were sacrificed. The lungs were harvested for biochemical analysis.

### Measurement of ferrous iron level and MDA level in lung tissues

Ferrous iron level in lung tissue was assessed using iron assay kit (DOJINDO, Kyushu, Japan) following the manufacturer’s instructions [23]. Relative malondialdehyde (MDA) concentrations in lung tissues were assessed using MDA content assay kit (Beyotime, Shanghai, China). Accurate calculations of ferrous iron and MDA levels were based on the total protein content of each sample, determined using the Enhanced BCA Protein Assay Kit (Beyotime, Shanghai, China) [24].

### Data acquisition and preprocessing of gene expression profile from preterm infants with BPD

Gene expression profiles from preterm infants with BPD were obtained from the datasets GSE8586 in the gene expression omnibus (GEO) database. The platform for GSE8586 was GPL570. GSE8586 provided microarray data of 54 umbilical cord tissue samples, including 20 from the preterm infants with BPD and 34 from preterm infants without BPD. All of the infants were born at 23 to 28 weeks gestational age from one of three centers (Brigham and Women’s Hospital, Beth Israel Deaconess Medical Center, and Wake Forest Medical Center). There were minimal differences in maternal characteristics between infants with and without BPD [25]. A total of 259 ferroptosis-related genes were downloaded from the FerrDb database (http://zhounan.org/ferrdb/legacy/). The data preprocessing was performed as follows: R package annotation was conducted to match probes and gene symbols. Probes with no mapped gene were removed, and multiple probes mapping to the same gene were calculated as the median value of the probes.

### Identification of differentially expressed genes

The R package EdgeR tool was used to analyze the differentially expressed genes between BPD and control samples from GSE8586 data set, with the criteria for significance as | logFC | > 1.5 and *p* < 0.05. These differentially expressed genes were shown in volcano plot and heat map.

### Functional enrichment analysis of differentially expressed genes

To explore the molecular interactions of differentially expressed genes, GO analysis, KEGG enrichment analysis and GSEA functional enrichment analysis were performed [26–28]. GO functional annotation analysis and KEGG enrichment analysis were performed using the package "Cluster Profiler" in R (version 4.0.5), with *p* value < 0.05 and false discovery rates (FDR) < 0.25 considered statistically significant.

### Identification of differentially expressed ferroptosis-related genes

Intersections of differentially expressed genes and ferroptosis-related genes were taken to obtain differentially expressed ferroptosis-related genes. Differentially expressed lncRNAs were dependent on the overlap of differentially expressed genes and lncRNAs. Correlation analysis between differentially expressed ferroptosis-related genes and differentially expressed lncRNAs was performed, and those genes with correlation coefficient *r* > 0.2 and *p*-value < 0.05 were determined as ferroptosis-related lncRNAs (FRLs).

### Identification and validation of diagnostic FRLs

To explore the diagnostic FRLs, random forest and LASSO regression were conducted based on the expression level of FRLs respectively. The intersection of candidates obtained from random forest and LASSO regression was chosen as the diagnostic FRLs. To assess the predictive accuracy of each diagnostic FRLs, the ROC analysis was conducted using “ROCR” R package.

### Constructing a predictive model based on diagnostic FRLs

Multivariate Cox regression analysis was used to model all the diagnostic FRLs, and nomograms and ROC curves were created to calculate the predictive power of this model. Furthermore, the model was internally validated using 40% of the samples, with the area under the ROC curve (AUC) calculated in addition.

### Calculation of ferroptosis-related lncRNA risk score

The ferroptosis-related lncRNA risk score (FRL score) was calculated as the sum of multiplication of coefficient and the standardized expression values of each diagnostic FRLs. Samples were subsequently stratified into high or low FRL score group based on the median value of FRL score. Gene set variation analysis (GSVA) scores of the KEGG pathway were performed for all the samples. The significant differences between immune-related and metabolic-related pathways in high and low FRS score group were displayed individually. GSVA scores for Hallmark gene sets were also calculated to explore the differences between high and low FRL score group.

### Collection of umbilical cord blood from preterm infants and term infants

In our study, 12 preterm infants born at 28 to 32 weeks gestational age and 20 term infants without small for gestational age and fetal distress in Clinical Ethics Committee of Maternal and Child Health Hospital of Guiyang City from March 2023 to April 2023 was selected as participants. Umbilical cord blood from these participants was collected in EDTA-containing vacutainer tubes. The Research Ethics Commission of Guiyang Maternal and Child Health Care Hospital approved the study. Informed consent was obtained from all subjects’ legal guardians. All methods were carried out in accordance with relevant guidelines and regulations. All experimental protocols were approved by Ethics Commission of Guiyang Maternal and Child Health Care Hospital.

### Umbilical cord blood lymphocytes purification

Whole blood was processed to obtain lymphocytes as follows. Briefly, 5 ml blood was diluted with 5 ml Phosphate Buffered Saline (PBS), then layered over 10 ml of Ficoll™ Paque Plus (GE Healthcare, Pittsburgh, PA, USA) in a 50 ml conical tube and spun at 2000 rpm for 30 min at room temperature. The buffy coat was collected and washed 3 times.

### RNA preparation, cDNA synthesis, and quantitative Real-Time PCR

Total RNA was extracted from the lung tissue and blood samples using RNA isolation kit (Axygen, Union City, USA) and was quantified using NanoDropTM Spectrophotometer technology (Thermo Fischer Scientific, Wilmington, DE, USA). RNA was reversely transcribed to cDNA using a reverse transcriptase kit (TaKaRa, Tokyo, Japan). The reversed cDNA was used for real-time qPCR with SYBR Green PCR Master Mix (TaKaRa, Tokyo, Japan) following the manufacturer’s protocols. Samples were assayed in triplicate. The internal reference gene was actin. The abundance of mRNA of target genes was determined by relative expression to the respective actin by the 2^−∆∆Ct^ method. All primers are shown in the Table 1.

**Table 1:** Primer sequences

Gene	Forward Primer Sequence	Reverse Primer Sequence
FTH	CATCATGACCACCGCGTCTC	AGTCATCACGGTCAGGTTTCTTT
PRKAA1	TTCGGGAAAGTGAAGGTGGG	GGTTCTGGATCTCTCTGCGG
ATM	CTTAAGGGTTCTCGTCGACCT	AACGTGCATCCTCACCTCAC
Tp53	CCCCTGAAGACTGGATAACTGT	CAGGAGCTGACACTTGGAGG
Atf3	GGAGCCGACCGACCAAC	TGAAGCATCATTTTGCTCCAGTC
Hmox	CTAAGACCGCCTTCCTGCTC	TGCAGAGGTAGTATCTTGAACC
Gss	GACAACGAGCGAGTTGGGAT	TGAATGGGGCATACGTCACC
Acsl4	CACCTTCGATCCCAGGAGATT	GAGCGATATGGACTTCCGGG
Actin Rat	CCGCGAGTACAACCTTCTT	TGAAGGTCTCAAACATGATCTGG
LINC01103	GGTGTGGGTAGAGCTTGTCC	GCTGCAGTTGCATGAATGGT
TTTY8	AGCAGCACGTCATACCCAAG	ACCCACCTTATTGCTGCTCA
PACRG-AS1	ACGTGTCTATCCCGGTCTCT	AGGTCATCCAAGCCTCTTGC
POT1-AS1	TGGCGAAATACTGACAGGATG	GCTCCAGGATAGACGGTTTG
LINC00691	CAGAGGAAGAGATGAGAAACGG	GGATGCGTGCTCTAGAATGAG
LINC01103	GCCGTTGTTGATGAATCTGGG	ACTGTAGTGATGGTGTCTGCG
Actin Human	GCAAATGCTTCTAGGCGGAC	TAACAACGCATCTCATATTTGGAA

### Statistical analysis

The statistical analysis in this study was carried out using software R version 3.6.1 and GraphPad Prism software. A *p*-value of 0.05 was set as the threshold of statistical significance. The differentially expressed genes were screened with standard of *p* value < 0.05 and | Log2 fold change (FC) | > 1. LASSO regression analysis was used to exclude highly correlated genes and prevent over-fitting. Spearman analysis was used to evaluate the correlations between genes. Between-group differences of continuous parametric variables were calculated using Student’s t-test.

## Results

### Overall flowchart of the study

**Fig. 1:**
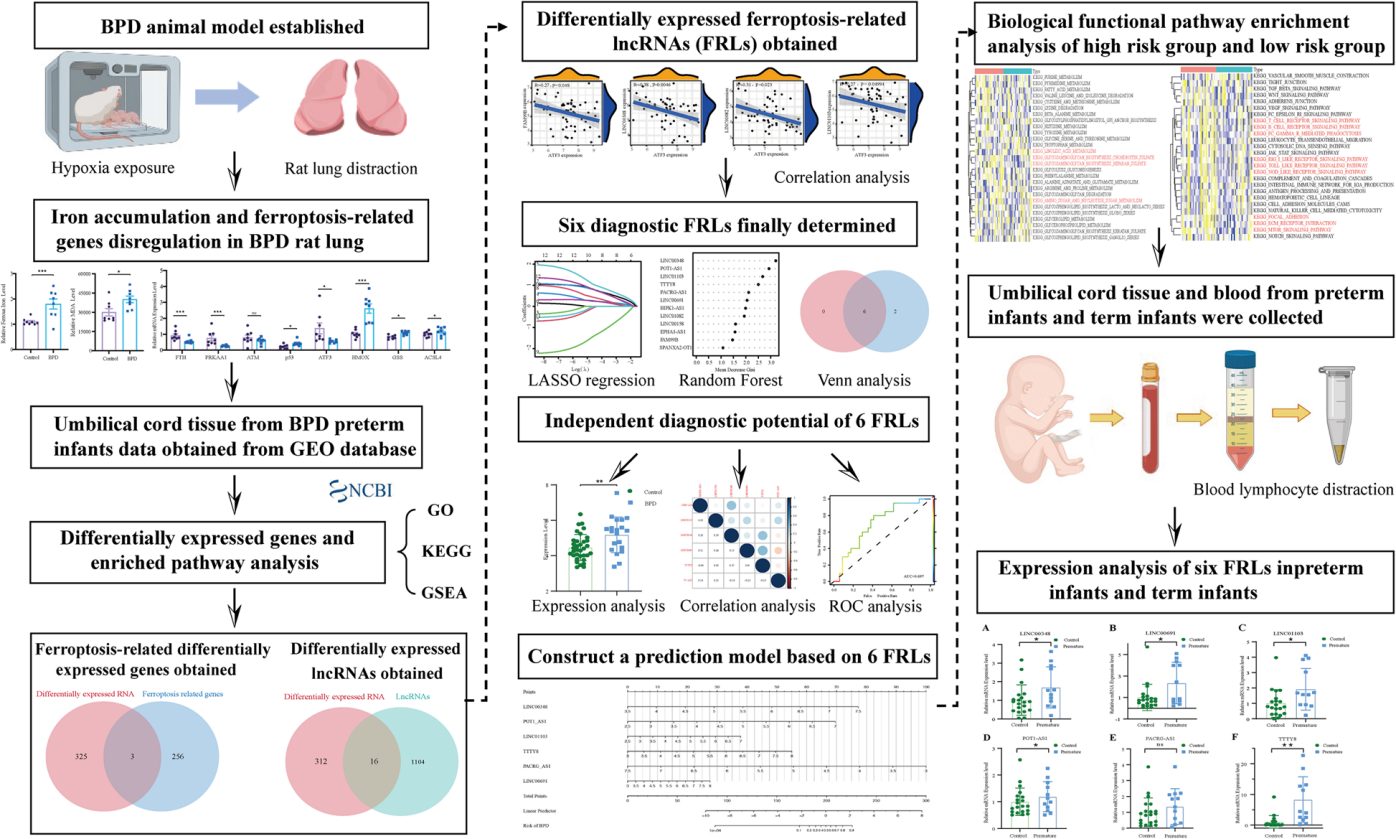
The flow diagram of this study. The study was divided into four parts: identification of elevated iron accumulation in BPD animal model, identification of biomarkers in GEO database associated with BPD, construction of a BPD predictive model, validation of FRLs expression level in preterm infants

The flowchart of our study was shown in Fig. 1. Ferrous iron accumulation and dysregulated ferroptosis-related genes expression were found in a BPD rat model, implicating ferroptosis participating in development of BPD. Next, the expression data from preterm infants with or without BPD was downloaded from GEO database. The differentially expressed genes were then identified and performed GO, KEGG and GSEA analysis. The differentially expressed genes were then intersected with lncRNAs and ferroptosis-related genes to get differentially expressed ferroptosis-related genes and differentially expressed lncRNAs. Correlation analysis was subsequently applied to identify differentially expressed ferroptosis-related lncRNAs (FRLs). Next, random forest and LASSO regression were performed individually. Based on intersections of two algorithms, 6 FRLs were finally chosen as diagnostic FRLs and constructed a BPD predictive model. KEGG, GO and hallmark analysis was applied to prove the potential function of the 6 FRLs. At last, umbilical cord blood lymphocytes from preterm infants and term infants were collected and made an mRNA expression validation.

### Elevated iron deposition and over-active ferroptosis in BPD rat lungs

**Fig. 2:**
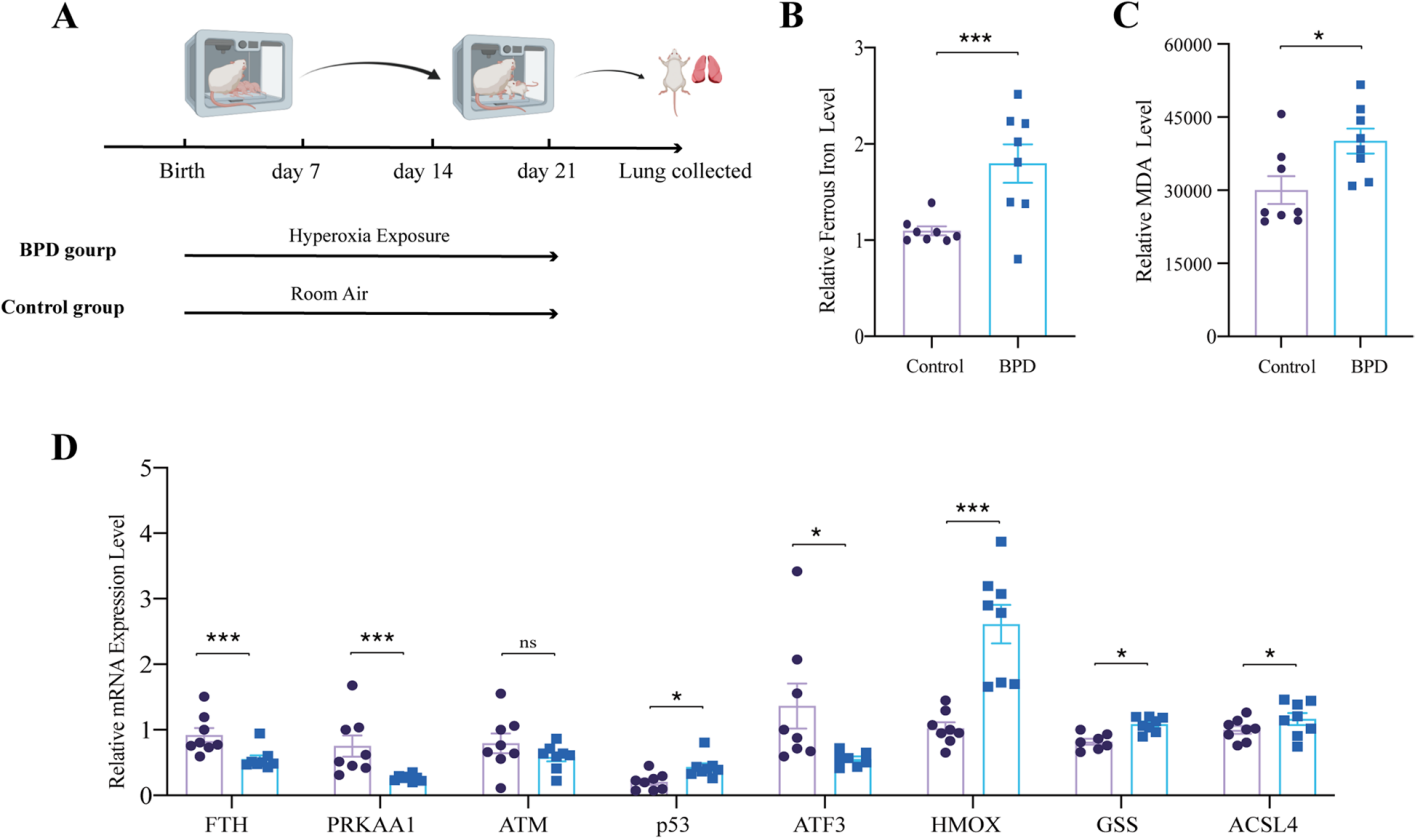
Elevated iron deposition and overactive ferroptosis in lungs from BPD rats. **A** Establishment of a rat BPD model. **B** Relative Fe^2+^ level in lungs of BPD and control rats. **C** Relative MDA level in lungs of BPD and control rats. **D** Relative mRNA expression level of ferroptosis-related genes in lungs of BPD rats and control rats. MDA, malondialdehyde, ACSL4, acyl-CoA synthase long-chain family member 4. HMOX1, heme oxygenase 1. FTH, ferritin heavy chain. ATM, ataxia telangiectasia mutated. ATF3, activating transcription factor 3. PRKAA1, protein kinase AMP-activated catalytic submit alpha-1. GSS, Glutathione. All data throughout the figure was presented as mean ± SEM (*n* ≥ 7). Statistical comparison by unpaired t-test, * *p* < 0.05, ** *p* < 0.01

Iron accumulation, together with lipid peroxides, plays a critical role in triggering ferroptosis [29]. An important indicator of lipid peroxidation is MDA, which is formed through enzyme catalyzed and chemical reactions involving polyunsaturated fatty acids [30]. To investigate the potential involvement of ferroptosis in the progression of BPD, we assessed the levels of ferrous iron and MDA in lung tissue distracted from BPD and control rats (Fig. 2A). As a result, the BPD rats exhibited significantly higher ferrous iron level and MDA level compared with control rats (Fig. 2B-C). We furtherly analyzed the mRNA expression level of ferroptosis-related genes, the results showed that, compared to those from control rats, the mRNA level of p53, heme oxygenase 1 (HMOX1), glutathione (GSS), Acyl-CoA synthase long-chain family member 4 (ASCL4) in lungs from BPD rat were upregulated, the mRNA levels of ferritin heavy chain (FTH), protein kinase AMP-activated catalytic submit alpha1 (PRKAA1) and activating transcription factor 3 (ATF3) were downregulated. There was no significant difference in the mRNA level of ataxia telangiectasia-mutated gene (ATM) between two groups. Given p53, HMOX1, ASCL4, GSS are the ferroptosis-promoting genes and FTH, PRKAA1 are the ferroptosis-inhibiting ones, ferroptosis may contribute to the progression of BPD (Fig. 2D).

### Differentially expressed genes in preterm infants with BPD

**Fig. 3:**
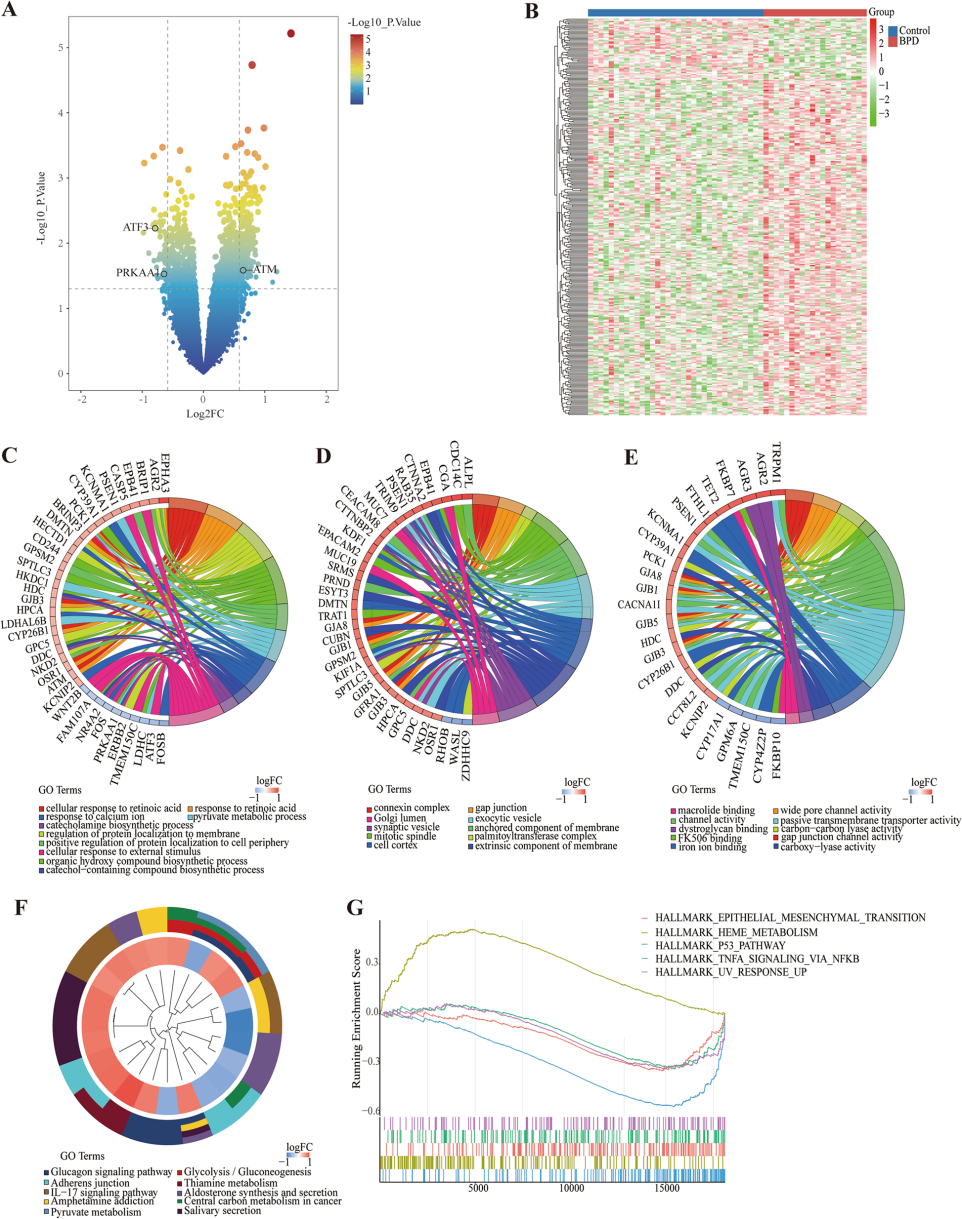
Differentially expressed genes in preterm infants with BPD. **A** Volcano plot of differentially expressed genes associated with BPD. **B** Heatmap of differentially expressed genes associated with BPD. **C** BP (**D**) CC (**E**) MF enrichment analysis of differentially expressed genes. **F** Enrichment analysis of KEGG pathway for differentially expressed genes. **G** GSEA analysis of differentially expressed genes. BP, biological process. CC, cellular component. MF, molecular function. KEGG, Kyoto encyclopedia of genes and genomes. GSEA, gene set enrichment analysis

The promising results of animal experiments inspired us to further analyze the microarray dataset to explore the role of ferroptosis in preterm infants with BPD. We firstly downloaded the gene expression profile (GSE8586) from the GEO database, which contained RNA-seq data of umbilical cord tissue from 20 preterm infants with (BPD group) and 34 preterm infants without BPD (control group). All of the infants were born at 23 to 28 weeks gestational age. A total of 328 differentially expressed genes (277 up-regulated and 51 down-regulated) were determined and displayed in volcano plot and heatmap plot individually (Fig. 3A-B). The differentially expressed genes were furtherly analyzed by GO functional enrichment, including biological process (BP), cellular component (CC), and molecular function (MF). BP were mainly enriched in *cellular response to retinoic acid, response to retinoic acid,* and *regulation of protein localization to the membrane* (Fig. 3C). CC analysis revealed that *connexin complex, gap junction,* and *palmitoyl transferase complex* were dominant (Fig. 3D). MF were largely enriched *in the connexin complex, gap junction,* and *palmitoyl transferase complex* (Fig. 3E). KEGG analysis were also performed, displaying a central enrichment in the *Glucagon signaling pathway, Glycolysis / Gluconeogenesis,* and *Amphetamine addiction* (Fig. 3F). In addition, GSEA enrichment analysis showed that differentially expressed genes were positively correlated with *epithelial-mesenchymal-transition, heme-metabolism, p53-pathway* and *UV-response-up* (Fig. 3G).

### Differentially expressed ferroptosis-related lncRNAs (FRLs) in preterm infants with BPD

**Fig. 4:**
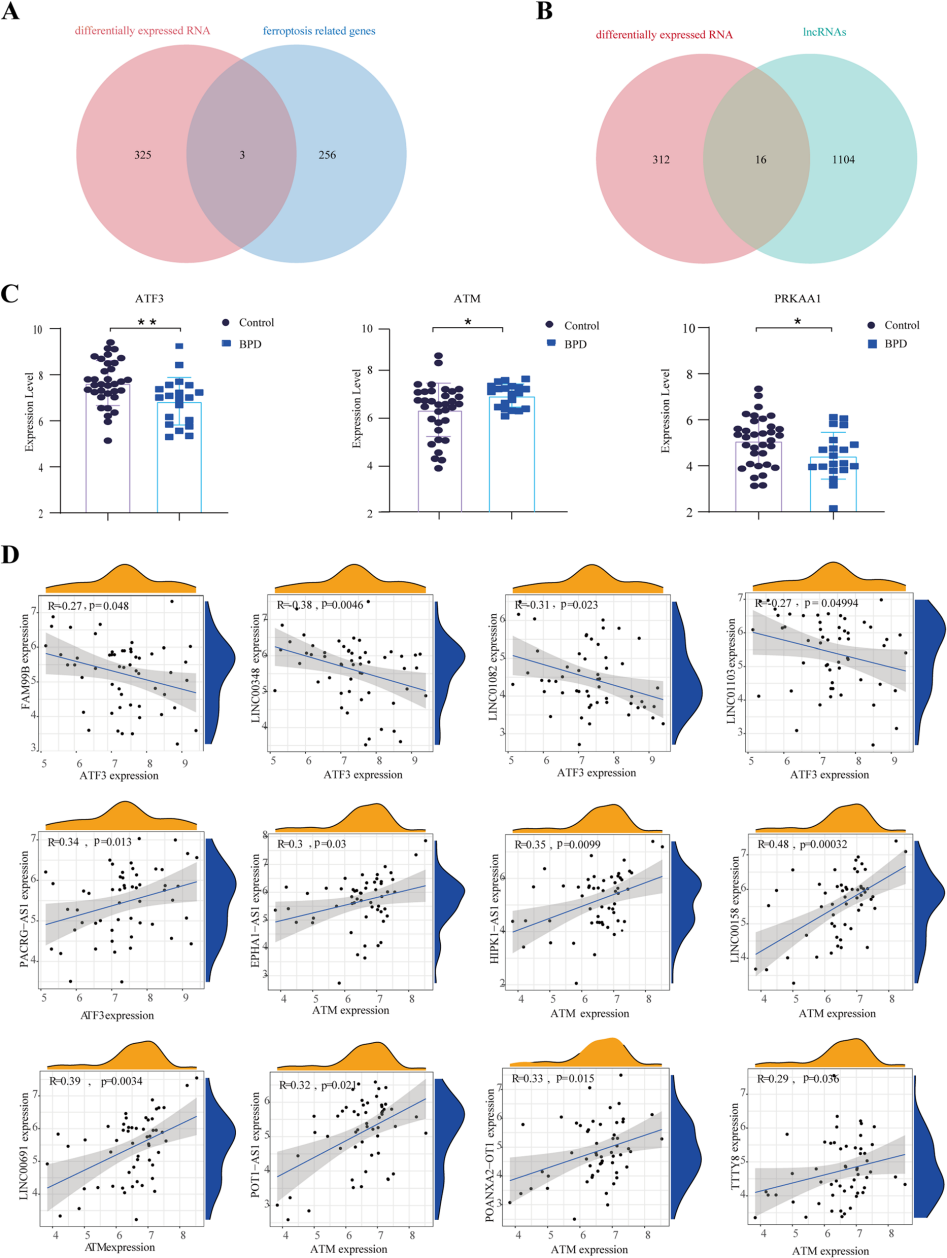
Differentially expressed ferroptosis-related lncRNAs (FRLs) in preterm infants with BPD. **A** Venn diagram to identify 3 differentially expressed ferroptosis-related genes. **B** Venn diagram to explore 16 differentially expressed lncRNAs. **C** Expression analysis of 3 differentially expressed ferroptosis-related genes in dataset. **D** Correlation analysis to determine 12 FRLs

By intersecting the differentially expressed genes with a set of 259 ferroptosis-related genes, three ferroptosis-related differentially expressed genes, namely ATF3, PRKAA1, and ATM, were identified (Fig. 4A). In the BPD group, the mRNA levels of PRKAA1 and ATF3 were downregulated, with that of ATM upregulated, when compared with control group (Fig. 4C). The variations of PRKAA1 and ATF3 in BPD group were in accordance with those in BPD rats, furtherly demonstrating the reliability of our analysis. Meanwhile, to verify the differentially expressed lncRNAs, the intersection of differentially expressed genes and 1120 lncRNAs were performed, and 16 differentially expressed lncRNAs, namely EPHA1-AS1, FAM99B, HIPK1-AS1, LINC00158, LINC00348, LINC00461, LINC00691, LINC01082, LINC01103, LINC01405, PACRG-AS1, POT1-AS1, SLC25A5-AS1, SPANXA2-OT1, TTTY8 and VWA8-AS1, were determined, (Fig. 4B). Using correlation analysis between ferroptosis-related genes and differentially expressed lncRNAs, 12 differentially expressed ferroptosis-related lncRNAs (FRLs) were finally identified (Fig. 4D).

### Diagnostic FRLs in preterm infants with BPD

**Fig. 5:**
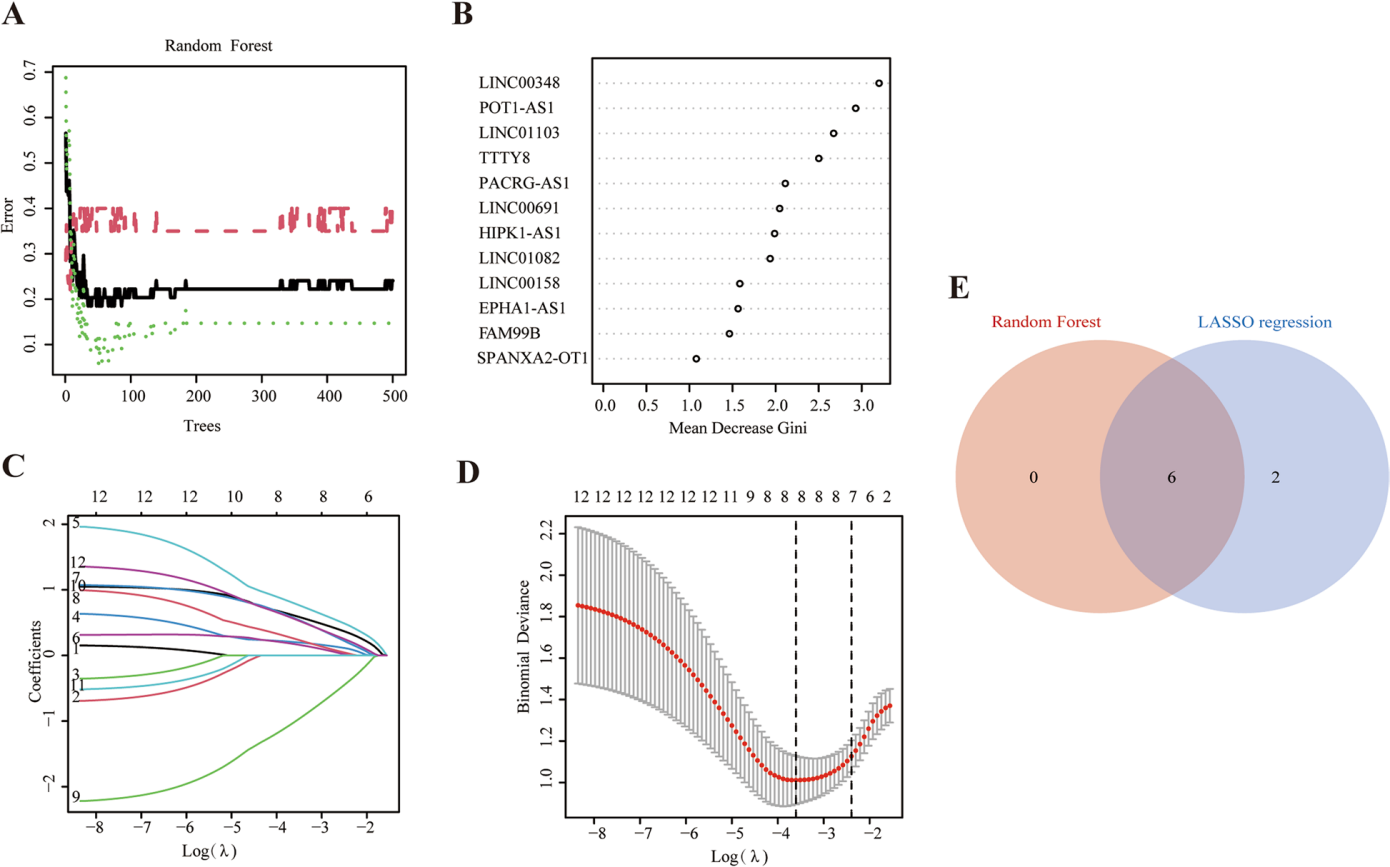
Diagnostic FRLs in preterm infants with BPD. **A-B** 6 diagnostic FRLs were determined using the random forest classifier Gini coefficients algorithm. **C** 8 diagnostic FRLs were obtained using LASSO regression. **D** Optimal λ selection in the LASSO regression. **E** Intersection results of random forest and LASSO regression, with a total of 6 diagnostic FRLs finally screened out. LASSO, least absolute shrinkage and selection operator

To narrow the number of diagnostic FRLs for BPD, two machine learning algorithms, random forest and LASSO, were performed on the 12 FRLs individually. In the random forest, the number of branches was suggested 35 to make minimum residual (Fig. 5A). According to the Gini coefficient, which determined the dimensional importance value of the random forest model, 6 FRLs (Gini coefficient > 2) were selected (Fig. 5B). In the LASSO regression, using the optimal penalty parameter (λ = 0.027), 8 FRLs were identified (Fig. 5C-D). The results of random forest and LASSO regression were intersected and a total of 6 FRLs were finally determined as the diagnostic FRLs for subsequent modeling. The 6 diagnostic FRLs were LINC00348, POT1-AS1, LINC01103, TTTY8, PACRG-AS1 and LINC00691 (Fig. 5E).

### Independent predictive potential of diagnostic FRLs

**Fig. 6:**
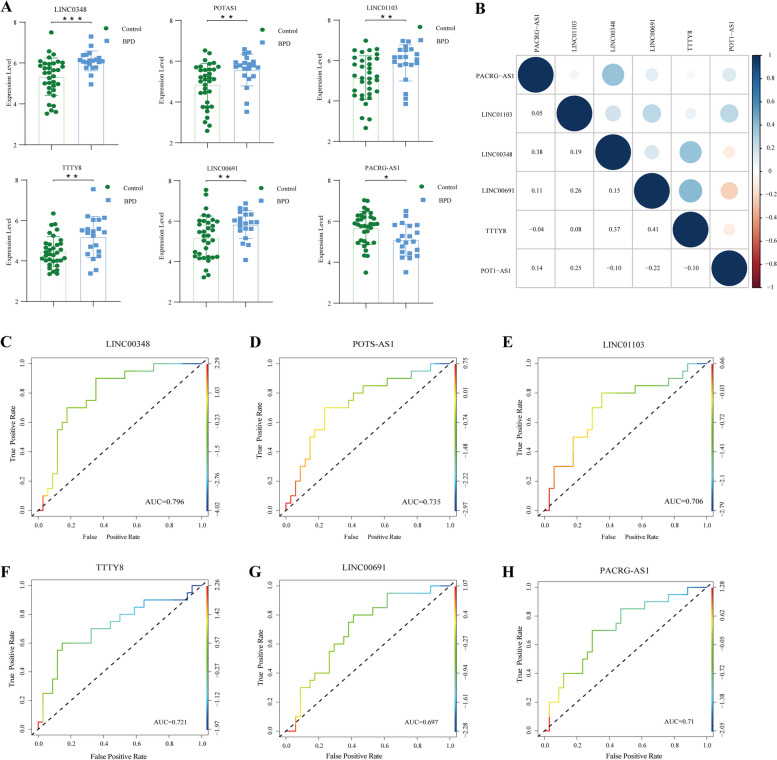
Independent predictive potential of diagnostic FRLs. **A** Expression analysis of 6 diagnostic FRLs between BPD group and control group. Data was presented as mean ± SEM. Statistical comparison by unpaired t-test, * *p* < 0.05, ** *p* < 0.01. **B** Correlation analysis between 6 diagnostic FRLs. Red and blue indicated positive and negative correlations respectively. **C** ROC curve of LINC00348 (AUC = 0.796). **D** ROC curve of POT1-AS1 (AUC = 0.736). **E** ROC curve of LINC01103 (AUC = 0.706). **F** ROC curve of TTTY8 (AUC = 0.721). **G** ROC curve of LINC00691 (AUC = 0.697). **H** ROC curve of PACRG-AS1 (AUC = 0.71). ROC, receiver operating characteristic. AUC, area under the ROC curve

The expression levels of the 6 diagnostic FRLs exhibited statistically significant differences between BPD and control group. Among them, five were upregulated in BPD group and regarded as “risk” signatures (Fig. 6A). Correlation analysis revealed mostly positive correlations among these FRLs, except for POT1-AS1, which showed negative correlations with LINC00348, LINC00691, and TTTY8 (Fig. 6B). To evaluate the predictive value of 6 diagnostic FRLs, individual ROC curves were plotted. The results showed that the area under the ROC curve (AUC) for each FRL ranged from 0.696 to 0.796, indicating their potential as diagnostic biomarkers of BPD (Fig. 6C-H).

### Constructing a predictive model based on 6 diagnostic FRLs

**Fig. 7:**
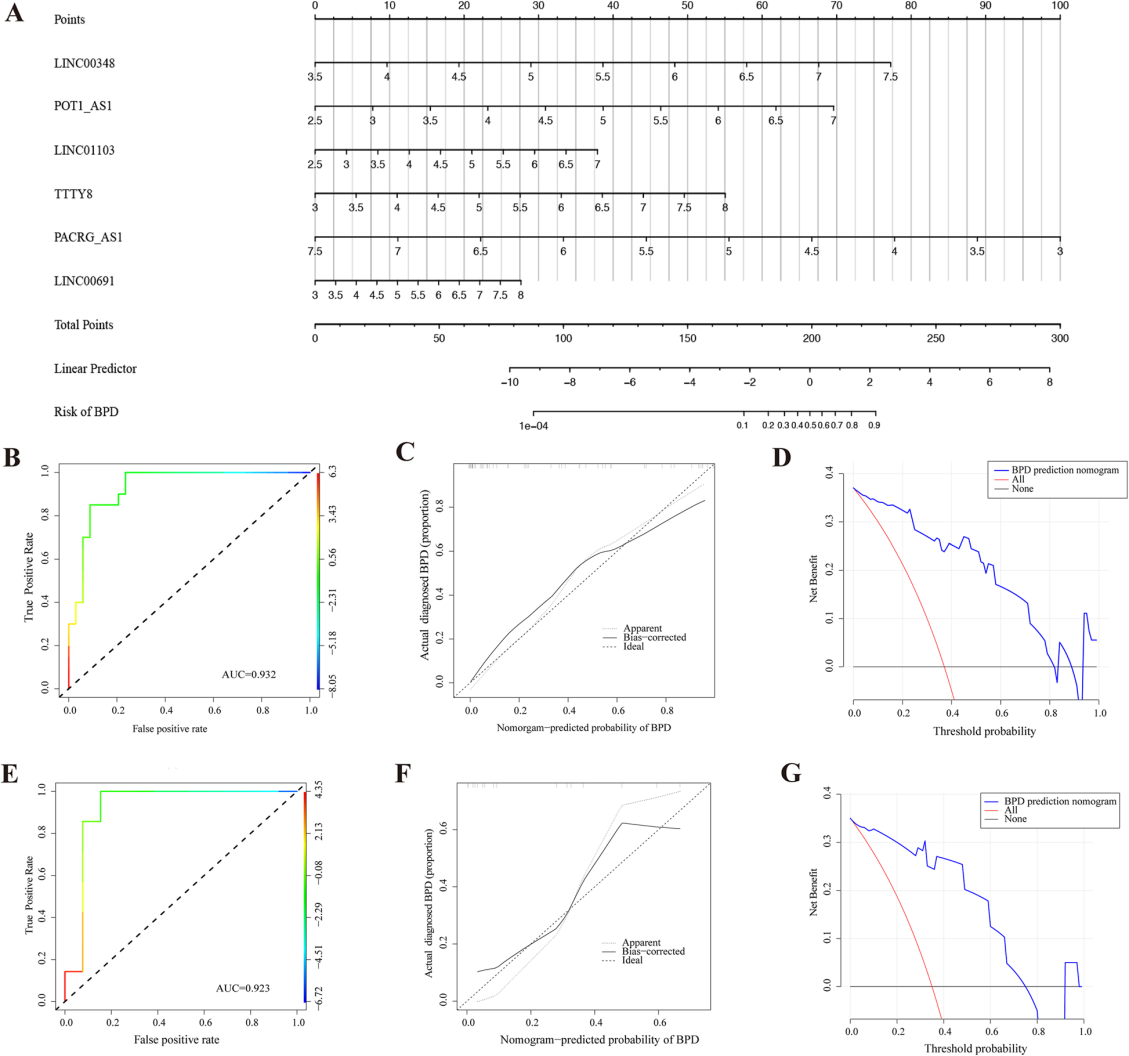
Constructing a predictive model based on diagnostic FRLs. **A** The predicting nomogram of 6 diagnostic FRLs for BPD incidence. **B** ROC curve for the training set (AUC = 0.932). **C** Calibration curve for the training set (mean absolute error = 0.053 mean squared error = 0.00393). **D** DCA curve for the training set. **E** ROC curve for internal validation (AUC = 0.923). **F** Calibration curve for internal validation (mean absolute error = 0.046 mean squared error = 0.00328). **G** DCA curve for internal validation. ROC, Receiver operating characteristic. AUC, area under the ROC curve. DCA, decision curve analysis

In univariate analysis, all 6 FRLs showed significant associations with the risk of BPD, supporting the establishment of a diagnostic nomogram model based on these FRLs. The nomogram calculated the final risk of BPD by summing up the scores assigned to each FRL. For instance, if the detection result of LINC00348 is 7, the corresponding score is 67.5, and similar scores are calculated for the others. A total score which exceeds 225 indicated a BPD risk higher than 90%. Our nomogram indicated that POT1-AS1 was the most effective predictor, and the others contributing significantly as well (Fig. 7A). To assess the predictive value of the risk model, the dataset was classified randomly into training set and validation set. The satisfied predictive potential of the model was indicated by the high area under the ROC curve (AUC = 0.932) (Fig. 7B). Calibration plots of the training set demonstrated a high level of consistency with the actual outcome (Fig. 7C). The decision curve analysis (DCA) curve of the training set showed a satisfactory clinical net benefit (Fig. 7D). Consistent with the results of training group, the AUC value for validation group (AUC = 0.923) was also high (Fig. 7E). The calibration curves (mean absolute error = 0.046, mean squared error = 0.00328) and DCA curves of validation dataset further unveiled satisfactory performances (Fig. 7F-G). In summary, the model constructed by the 6 FRLs demonstrated precise prediction value for BPD.

### Biological functional and pathway enrichment analysis of high FRL score group and low FRL score group

**Fig. 8:**
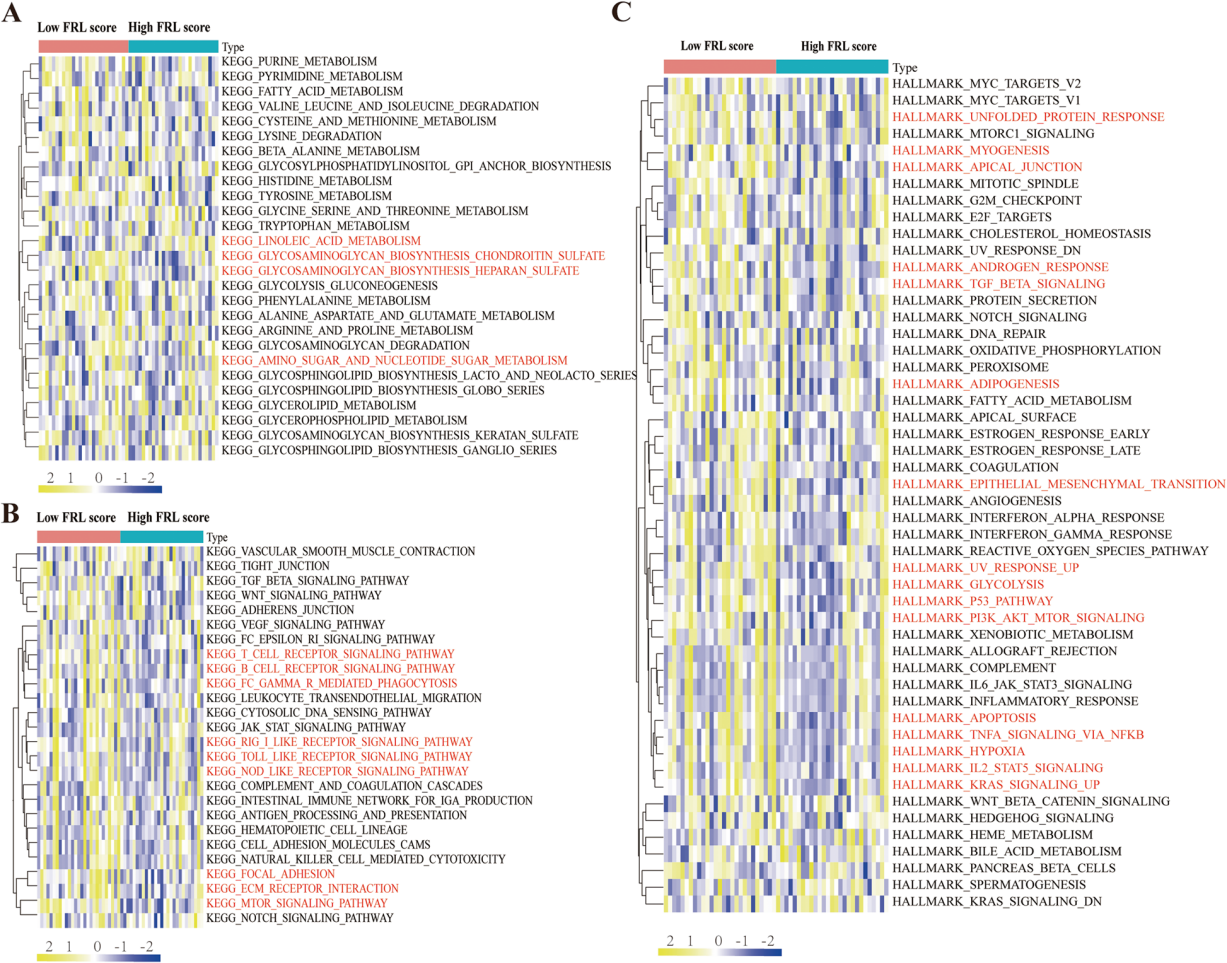
Biological functional and pathway enrichment analysis of high FRL socre group and low FRL score group. **A** Differences in GSVA scores of metabolism-related KEGG signaling pathway between high FRL score group and low FRL score group. **B** Differences in GSVA scores of immune-related KEGG signaling pathways between the high FRL score group and low FRL score group. **C** Differences in hallmarks between high FRL score group and low FRL score group. GSVA, gene set variation analysis. FRL score, scores of ferroptosis-related lncRNAs

Subsequently, we calculated FRL score for each sample based on the expression levels of 6 FRLs. The samples were then divided into high FRL score group and low FRL score group. To explore the physiological function and signaling pathways between high FRL score group and low FRL score group, hallmark pathway and KEGG analysis were conducted via gene sets variation analysis (GSVA). The results showed that, metabolism related KEGG pathway had significant differences on *the Linoleic acid metabolism, glycosaminoglycan biosynthesis-chondroitin sulphate, glycosaminoglycan biosynthesis-heparan sulphate,* and *nucleotide sugar* (Fig. 8A).The immune-related KEGG pathways had significant differences on* T cell receptor signaling pathway, B cell receptor signaling pathway, Toll-like receptor signaling pathway, Nod-like receptor signaling pathwa*y and *ECM-receptor interaction pathway* between two groups (Fig. 8B). In addition, hallmark pathways were also significantly different in *Unfolded protein response, Myogenesis, apical junction, Androgen response, TGF beta signaling, Epithelial and mesenchymal transition, p53 pathway*, *apoptosis pathway* and *hypoxia pathway* (Fig. 8C). Several under-expressed pathways in BPD supported it a developmental disorder which had lifelong influence.

### Expression levels of 6 diagnostic FRLs in umbilical cord blood lymphocytes from preterm infants and term infants

**Fig. 9:**
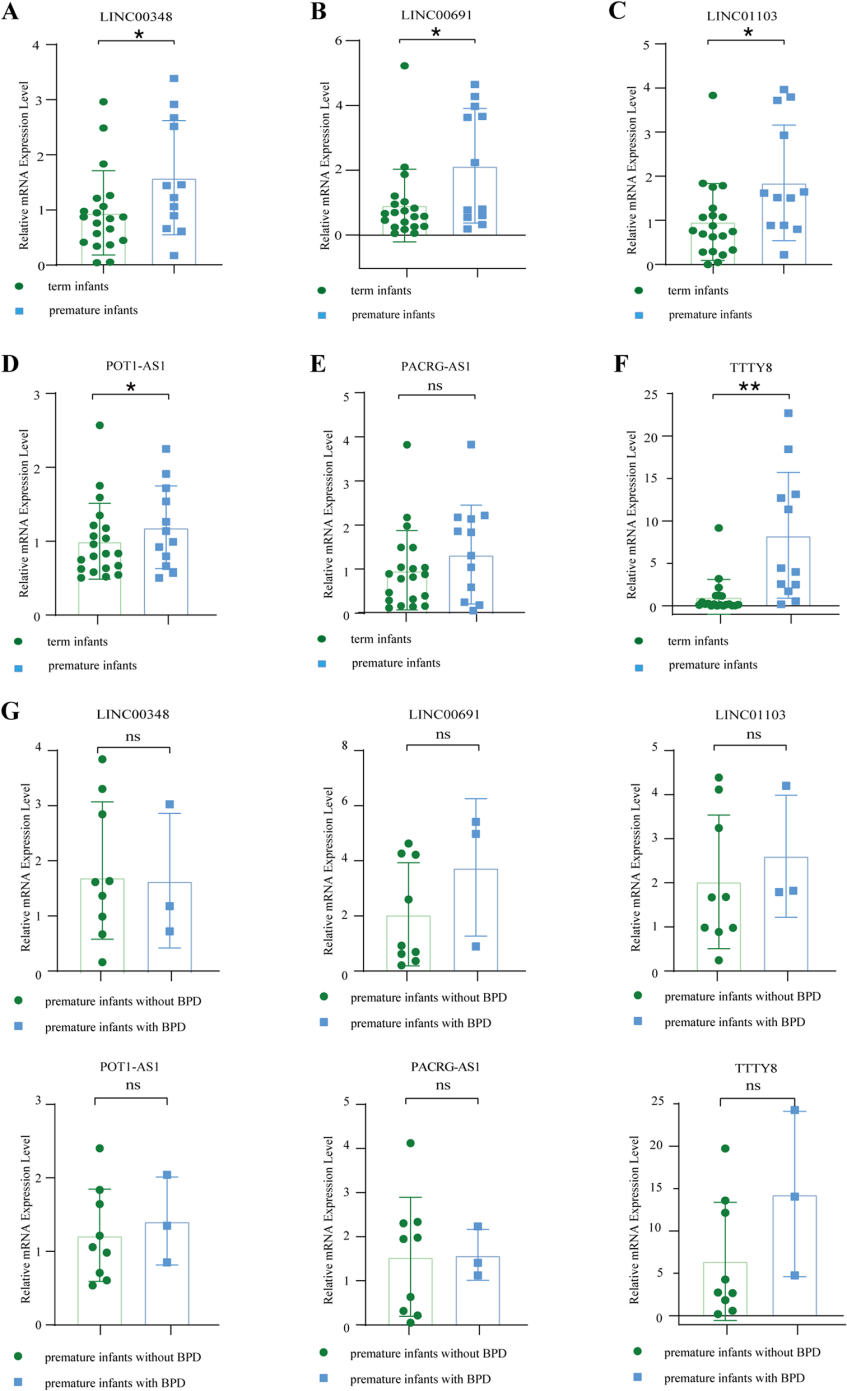
Expression levels of 6 diagnostic FRLs in umbilical cord blood lymphocytes from preterm infants and term infants. **A** Relative mRNA expression of LINC00348 in umbilical cord blood lymphocytes of preterm infants born before 32 weeks gestational age versus term infants. **B** Relative mRNA expression of LINC00691 in umbilical cord blood lymphocytes of preterm infants born before 32 weeks gestational age versus term infants. **C** Relative mRNA expression of LINC01003 in umbilical cord blood lymphocytes from preterm infants born before 32 weeks gestational age versus term infants. **D** Relative mRNA expression of POT1-AS1 in umbilical cord blood lymphocytes from preterm infants born before 32 weeks gestational age versus term infants. **E** Relative mRNA expression of PACRG-AS1 in umbilical cord blood lymphocytes from preterm infants born before 32 weeks gestational age versus term infants. **F** Relative mRNA expression of TTTY8 in umbilical cord blood lymphocytes from preterm infants born before 32 weeks gestational age versus term infants. **G** Relative mRNA expression of LINC00348, LINC00691, LINC01003, POT1-AS1, PACRG-AS1, TTTY8 in umbilical cord blood lymphocytes from preterm infants with BPD versus preterm infants without BPD. All data throughout the figure was presented as mean ± SEM (*n* = 3 to 20). Statistical comparison by unpaired t-test, * *p* < 0.05, ** *p* < 0.01, ns = no significance

Due to lung samples and peripheral blood from preterm infants are rarely available, we collected umbilical cord blood lymphocytes as a substitute. A total of 20 umbilical cord blood lymphocytes from term infants and 12 umbilical cord blood lymphocytes from preterm infants born before 32 weeks gestational age were collected. Consistent with our bioinformation analysis, the expression level of LINC00348, POT1-AS1, LINC01103, TTTY8, and LINC00691 were significantly upregulated in umbilical cord blood lymphocytes from preterm infants in comparison with those from term infants (Fig. 9A-F). In addition, a total of 3 infants eventually developed BPD among the 12 premature infants. A statistical analysis comparing the premature infants with BPD and the preterm infants without BPD were conducted in further. However, only a trend was observed due to the small sample size (Fig. 9G).

## Discussion

In our study, we found increased iron accumulation, upregulated MDA level, and several dysregulated ferroptosis-associated genes in lungs from BPD rats. These genes included p53, GSS, ATF3, FTH, HMOX1, ASCL4, PRKAA1 and ATM. Furthermore, by analyzing data of preterm infants with BPD, we identified 6 FRLs to construct a multivariate logistic regression model. The AUC of the ROC (0.932), internally validated ROC (AUC = 0.923), calibration, and DCA curves illustrated a good discrimination of BPD in preterm infants. Additionally, we observed increased expression level of LINC00348, POT1-AS1, LINC01103, TTTY8, and LINC00691 was increased in umbilical cord blood lymphocytes from infants born before 32 weeks gestational age, a population at higher risk of developing BPD. Taken together, the predictive model based on the 6 FRLs would help clinicians for early diagnosis of BPD.

Ferroptosis is a form of cell death triggered by iron overload or lipid peroxidation, resulting in the production of MDA as the primary end product. Given the challenges in directly accessing infants’ lung tissue, we explored the mRNA level of ferroptosis-related genes and ferrous iron accumulation in BPD rat lungs. The results exhibited increased free iron and MDA level in BPD rat lungs, with several ferroptosis-associated genes (p53, GSS, ATF3, FTH, HMOX1, ASCL4, PRKAA1, ATM) dysregulated. The expression level of ATF3 and PRKAA1 in preterm infant with BPD displayed similar trends as those in BPD rats, in turn verifying the accuracy of the bioinformatics analysis prediction in our study. HMOX1, the rate-limiting enzyme in heme degradation, stimulated ferritin synthesis and disturbed iron metabolism, thus leading to ferroptosis [31]. ASCL4, an enzyme involved in phospholipid metabolism, affects ferroptosis by catalyzing the formation of PUFA-CoA [32]. FTH increased cellular resistance to ferroptosis [33]. The inactivation of AMP-activated protein kinase, the protein encoded by PRKAA1, sensitized cells to ferroptosis [34]. ATM, the major sensor of DNA double-strand break damage, activated ferroptosis depending on downstream target p53 [35]. ATF3 regulated activities of p53 in addition [36]. Collectively, our results suggested that ferroptosis was involved in pathogenesis and development of BPD.

In our study, we identified 6 FRLs, namely LINC00348, POT1-AS1, LINC01103, TTTY8, PACRG-AS1 and LINC00691, which showed promise as biomarkers for BPD diagnosis. Previous studies reported that knocking down POT1-AS1 reduced cyclin D1 and cyclin-dependent kinase 4 protein expression, hindering progress of BPD [37, 38]. TTTY8 was found. to be upregulated in endometrium from patients with endometriosis, a condition that shared similar with BPD [39, 40]. Additionally, LINC00348 was implicated in endoplasmic reticulum stress apoptosis and autophagy, which were molecular mechanisms of cell death in BPD [41–43]. Besides, the inhibition of LINC00691 expression was shown to inhibit AKT activity [44]. Given a cross-talk between AMPK (the protein encoded by PRKAA1) and AKT [45], LINC00691 may regulate progression of BPD through AMPK pathway. Overall, these findings indicate the potential involvement of these FRLs in the progression of BPD and highlight the relevance as diagnostic markers.

For a model, the AUC value larger than 0.9 indicates a high discrimination, but clinical prediction models for BPD often struggle to achieve an AUC of ROC greater than 0.8 [46]. In our study, the AUC value of the predictive model constructed based on 6 FRLs was 0.932, illustrating a good discrimination in preterm infants with BPD. Furthermore, the variations of FRLs’ expression level in umbilical cord blood from preterm infants were consistent with the bioinformatics analysis prediction. As a surrogate for lung tissue, the whole blood-derived mononuclear cells gene expression profiling holds potential to provide novel biomarkers for BPD diagnosis [47]. And epigenetic modifications in leukocytes partially reflect similar changes in lung tissue [48, 49]. Thus, FRLs in blood lymphocytes, which is one type of the epigenetic changes, may serve as potential biomarkers for predicting the risk of BPD.

It is worth noting that ferroptosis is not only happened in endothelial cells, but also lymphocytes [50]. Various types of immune cells, including NK cells, CD4^+^ T cells, CD8^+^ T cells, and Treg cells, are predicted to send signals to other cell types in the lung and lead to signaling cascades associated with hyperoxia [51]. We speculate that lymphocyte ferroptosis may release dangerous signals, such as reactive oxygen species (ROS). These signals activate the innate immune system and trigger an inflammatory reaction, ultimately leading to lung injury. Moreover, lymphocyte ferroptosis, coupled with inflammatory response, may further contribute to lung injury by arresting the development of lung [52].

Inevitably, there were still some limitations in our study. Blood markers may not fully reflect the specific cellular changes due to differences in cell lineage, and sorting umbilical vein endothelial cells would be a better alternative. In addition, further validation and clinical studies are necessary before implementing this predictive model in a real-world clinical setting.

In summary, using data from GEO database, BPD animal model and preterm infants blood sample, we constructed a ferroptosis-related predictive model and would help early diagnosis of BPD.

## Data Availability

Publicly available datasets were analyzed in this study. This data can be found as follow: https://www.ncbi.nlm.nih.gov/geo/,with the accession number GSE8586. The other data used during the current study would be available from the corresponding authors on reasonable request.
